# Possible interactions between gut microbiome and division of labor in honey bees

**DOI:** 10.1002/ece3.11707

**Published:** 2024-08-27

**Authors:** Kang Wang, Ming Zheng, Minqi Cai, Yi Zhang, Yuanchan Fan, Zheguang Lin, Zhi Wang, Qingsheng Niu, Ting Ji

**Affiliations:** ^1^ College of Animal Science and Technology Yangzhou University Yangzhou China; ^2^ Key Laboratory for bee Genetics and Breeding Jilin Provincial Institute of Apicultural Sciences Jilin China

**Keywords:** division of labor, gut microbiome, honey bee, pollen

## Abstract

Recent studies have provided new insights into the role of the microbiome in shaping host behavior. However, the relationship between the temporal division of labor among honey bees (*Apis mellifera*) and their gut microbial community has not been widely studied. Therefore, we aimed to evaluate the link between the gut microbiome and division of labor in honey bees by examining the microbial absolute abundance and relative composition of 7‐day‐old nurse bees and 28‐day‐old forager bees from a natural hive, as well as those of worker bees of the same 14‐day‐old age showing different behaviors in a manipulated hive. We found that forager bees had fewer core bacteria, particularly gram‐positive fermentative genera such as *Lactobacillus* and *Bifidobacterium*, with *Bifidobacterium asteroides* being the most sensitive to host behavioral tasks. Our results showed that forager bees have lower gut community stability compared to nurse bees, suggesting that their gut community is more susceptible to invasion by non‐core members. Furthermore, a pollen limitation experiment using caged honey bees indicated that dietary changes during behavioral shifts may be a driving factor in honey bee microbial diversity. This study contributes to a greater understanding of the interaction between the gut microbiome and behavioral tasks and provides a foundation for future assays.

## Graphical abstract

This work utilized molecular genetic techniques to evaluate the link between the microbiome and behavior in bees.

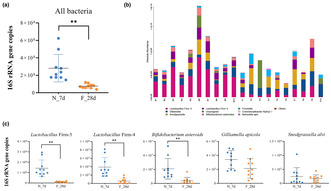

## 1 INTRODUCTION

Social behavior is one of the most complex evolutionary innovations allowing organisms to adapt to their environment, However, individuals often sacrifice their own development to enhance the environmental adaptability and competitive advantages of their colonies. Eusocial western honey bees (*Apis mellifera*) display a stereotyped but nutrition‐dependent plastic pattern of division of labor. Worker bees typically perform nursing behaviors inside the hive for the first 2–3 weeks of their life and then begin foraging outside the hive for nectar and pollen. It has been well documented that the age at which individual bees begin to forage is regulated by molecular and hormonal pathways that are related to individual nutritional status (Ament et al., 2010).

Recent studies have provided new insights into the role of the gut microbiome in shaping insect behavior. Multiple experiments involving *Drosophila melanogaster* have indicated that the gut microbiome is associated with host kin recognition (Lizé et al., 2014), social attraction (Venu et al., 2014), aggression behaviors (Jia et al., 2021), and mating preferences (Sharon et al., 2010). Metagenomic and 16S rRNA gene surveys revealed that honey bees harbor representatives of the same eight to nine bacterial species clusters. Five of the most prevalent clusters, *Lactobacillus* Firm4, *Lactobacillus* Firm5, *Snodgrassella alvi*, *Gilliamella apicola*, and *Bifidobacterium asteroides*, are considered the core microbiota of the honey bee gut (Kwong & Moran, 2016). The influence of gut microbes on the gut–brain axis has also been observed in honey bees. Gut microbes induce more successful learning and memory behavior (Cabirol et al., 2023; Zhang et al., 2022), and influence honey bee socialization by increasing the rate of head‐to‐head social interactions (Liberti et al., 2022). These studies demonstrate substantial bidirectional communication between the gut and brain and highlight the contributions of specific gut microbes to honey bee neurological and behavioral processes.

We examined the microbial absolute abundance and relative composition of nurse and forager bees in both natural and manipulated hives to elucidate the role of the gut microbiome in the honey bee division of labor. Additionally, we conducted a pollen limitation experiment to verify the hypothesis that differences in dependency on pollen nutrition between nurse and forager bees contribute to the different gut microbial characteristics observed in these two types of worker bees. These findings align with recent studies highlighting the influence of the microbiome on host behavior, particularly in relation to the temporal division of labor among honey bees. For example, Vernier et al. (2024) provide insights into the interaction between gut microbiota and behavioral tasks, reinforcing the relevance of our research. The results of this study provide a valuable reference for future in vivo assays and suggest a possible interaction between the gut microbiome and behavioral tasks in honey bees.

## 2 MATERIALS AND METHODS

### 2.1 Collection of natural nurse and forager honey bees

Natural nurse and forager bees were collected based on two criteria: age and behavior. We placed three brood combs with late‐stage pupae into a laboratory incubator. As the worker bees emerged, they were individually marked with colored spots on their thorax. Over 500 one‐day‐old marked honey bees were then reintroduced to the natural hive. We used a queen cell egg‐laying and rearing device to facilitate observation of workers feeding larvae in queen cells (Wu et al., 2015). On Day 7, the queen cell frame was placed in an observation box, and marked workers who frequently put their heads inside the queen cells to contact larvae were identified as nurse bees (Seeley, 1982). On Day 28, the entrance of the hive was blocked and marked workers who carried pollen were designated as forager bees. The entire guts of 10 natural nurse bees and 10 forager bees were dissected to analyze bacterial abundance and composition.

### 2.2 Collection of nurse and forager honey bees of the same age

To enable young bees to rapidly establish a division of labor, we built a composite hive following Amdam's hive manipulation method of removing natural foragers (Amdam et al., 2005). Generally, older foragers can return to their hive via the original entrance after foraging missions or move to a new environment due to their pre‐acquired memory of the hive site (Hammer & Menzel, 1995). In contrast, inexperienced nurse bees perform poorly in returning to the hive after being relocated. More than 500 one‐day‐old newly emerged bees were marked and then reintroduced to the original hive. On Day 7, the older foragers were removed (Amdam et al., 2005), and the remaining colony, with predominantly young bees, a queen, larvae, and food, was taken to a new location. On Day 14, we collected marked worker bees that showed nursing or foraging behaviors, as described in Section 2.1. Ten replicates from each behavioral group were used for honey bee gut bacterial characteristics analysis. This experiment was replicated with a different hive using the same methods.

### 2.3 Collection of honey bees fed different amounts of pollen

We propose a hypothesis that variations in dependency on pollen nutrition between nurse and forager bees may contribute to the distinct gut microbial characteristics observed in these two types of worker bees. To investigate this, newly emerged sterile bees were generated and randomly divided into three groups of 30 bees each, as described by Powell et al. (2014). The bees were fed pollen mixed with homogenates of natural nurse bee hindguts for 1 day to inoculate them with bacteria. Subsequently, the pollen feed was removed from the cages, and one group received no additional pollen (Group 1d), while the other two groups were fed sterilized pollen for either 4 (Group 5d) or 9 (Group 10d) days. On Day 10, the entire guts of 10 individual honey bees from each group were dissected for further analysis. This experiment was replicated with a different hive using the same methods.

### 2.4 Quantitative polymerase chain reaction and 16S amplicon sequence analysis

Total DNA was extracted from the entire gut according to the manufacturer's instructions (Vazyme, China). Total copies of the 16S rRNA gene were amplified using universal bacterial primers (F: 5′‐AGGATTAGATACCCTGGTAGTCC‐3′, R: 5′‐YCGTACTCCCCAGGCGG‐3′) (Du et al., 2021). Reactions (20 μL) were carried out in triplicate with 10 μL iTaq Universal SYBR Green (Vazyme, China), 0.4 μL (each) 10 μM primer, 5.2 μL H2O, and 4 μL of template DNA that had been diluted 70×. The PCR cycle was 95°C (3 min) followed by 35 cycles of 95°C (15 s), 60°C (15 s), and 72°C (1.5 min). Each plate contained a negative control with water as the template. Using standard curves from the amplification of the cloned target sequence in a pGEM‐T vector (Promega, US), we calculated the absolute copy number for the reaction template and then adjusted these values based on dilution to determine the total copy number for each sample. Based on the bacterial load measured using quantitative polymerase chain reaction, the absolute abundance of each species was calculated by multiplying the total load of the 16S rRNA genes by the percentage of relative abundance (Motta et al., 2018).

To analyze the gut microbiome composition, we amplified the V4 region of the 16S rRNA gene with common primer pairs. Quality filtering and microbiome composition analysis were performed as described (Hall & Beiko, 2018). Operational taxonomic units were clustered at a similarity threshold of 97%. A local database dedicated to the 16S rDNA sequences of honey bee gut bacteria was used for taxonomic assignment (Du et al., 2021).

### 2.5 Statistical analysis

Statistical tests were performed using the Wilcoxon rank‐sum test in SPSS, including the absolute and relative bacterial abundances. Adonis tests were performed to analyze the clustering between groups based on unweighted UniFrac distances. Significance was determined at *p* < .05, *p* < .001, and *p* < .0001.

## 3 RESULTS

### 3.1 Microbial characteristics of natural worker bees with different tasks

**FIGURE 1:**
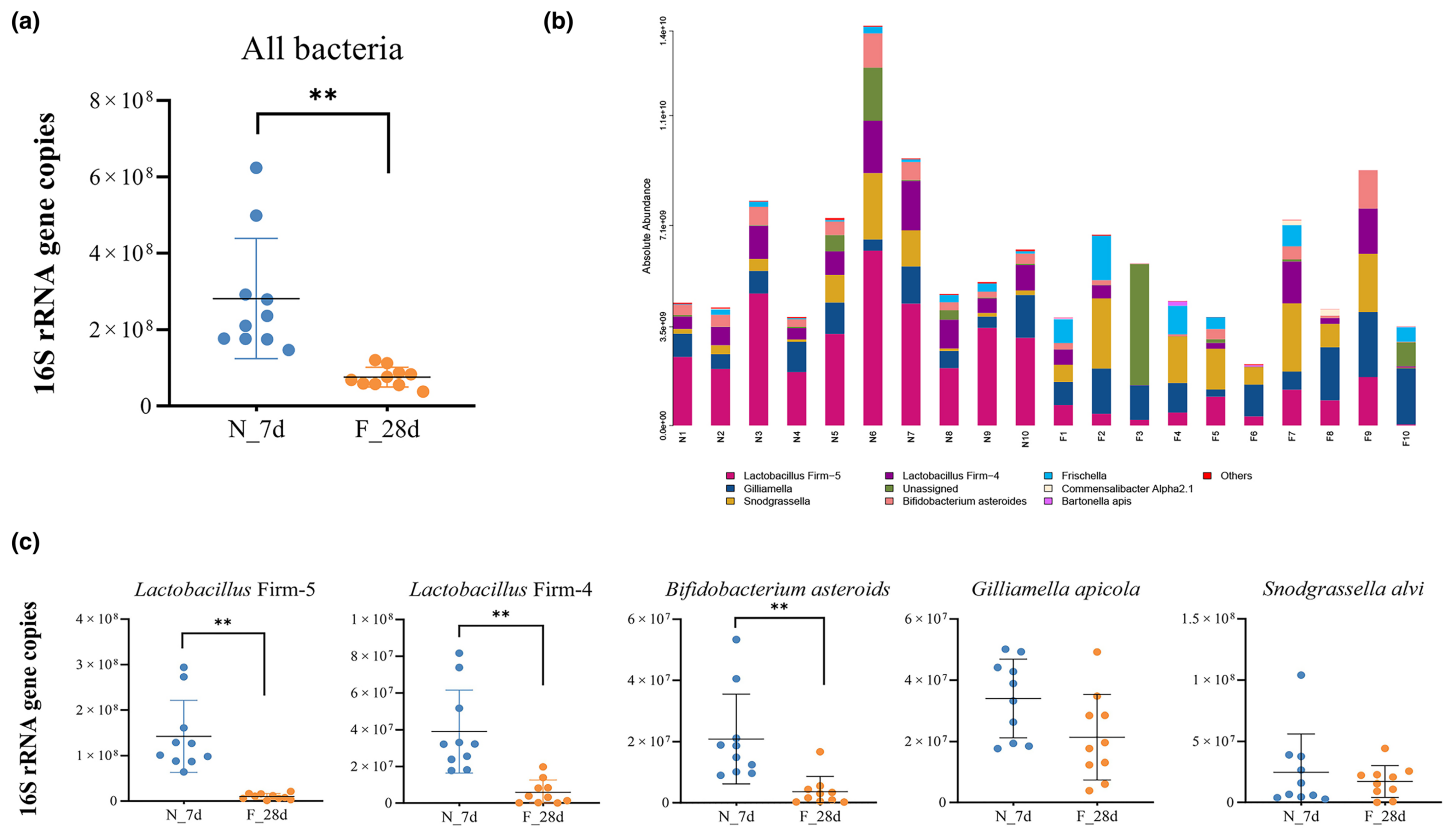
Honey bee gut microbiome abundance in natural nurse and forager bees. (a) and (b) Total 16S rRNA gene copies from all bacteria. N_7d: 7‐day‐old nurse bees; F_28d: 28‐day‐old forager bees. (c) Absolute copy numbers of five core bacterial species. Forager bees had lower total and core bacterial abundances. Significance was set at **p < .01.

**FIGURE 2:**
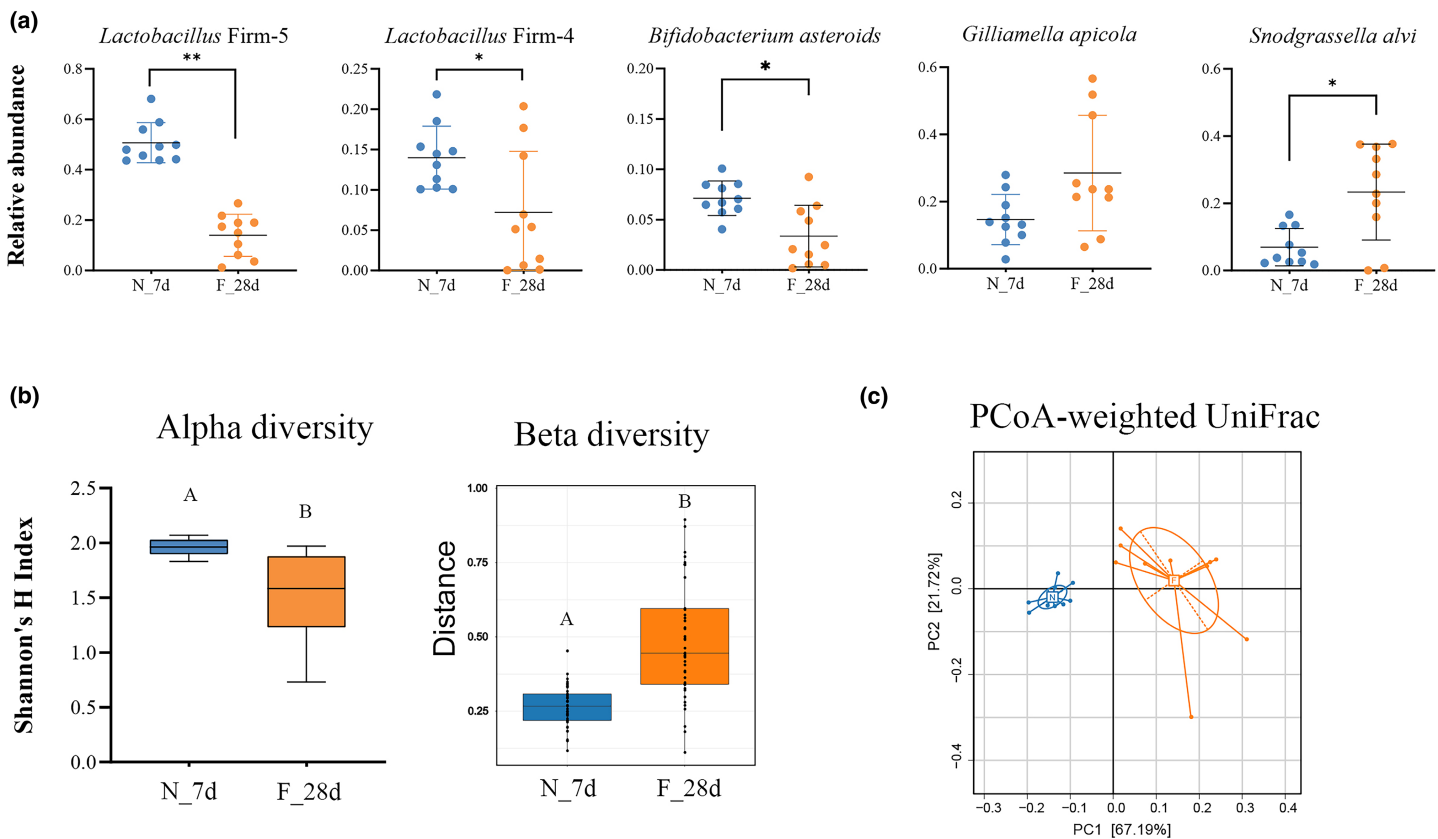
Honey bee gut microbiome composition in natural nurse and forager bees. (a) Relative abundances of the five core bacterial species. (b) Differences in alpha and beta diversities between the gut microbiomes of the two types of honey bees. (c) Principal coordinate analysis (PCoA) using weighted UniFrac distances. Significance was set at *p < .05, **p < .01.

To characterize the gut microbiota of worker bees with different social behavioral traits, we analyzed the total bacterial abundance and the individual abundance of five core species of bacteria (*L*. Firm 5, *L*. Firm 4, *B. asteroides*, *S. alvi*, and *G. apicola*) in natural workers performing nursing or foraging behaviors. A lower abundance of total bacteria was observed (Figure 1a,b) in forager bee guts compared to that in nurse bee guts (Wilcoxon test, *p* < .01). Specifically, there were 5‐ to 15‐fold lower levels of three core species (*L*. Firm 5, *L*. Firm 4, and *B. asteroides*) in forager bees compared to the levels in nurse bees (Wilcoxon test, *p* < .05) (Figure 1c). We further analyzed the relative composition of the gut community in each honey bee type. The three core species, *L*. Firm 5 (Wilcoxon test, *p* < .01), *L*. Firm 4, and *B. asteroides* (Wilcoxon test, *p* < .05), were also observed at lower levels of relative abundance in forager bees than in nurse bees (Figure 2a). Notably, the relative level of *S. alvi* was significantly higher in forager bees compared to that in nurse bees (Wilcoxon test, *p* < .05), which may have been due to the drop in the load of other species. All core species were detectable and dominant in both nurse and forager bee guts. However, in forager bees, the five core species were less dominant than they were in nurse bees (93.42% in nurse bees compared to 77.49% in forager bees) and were more likely to be supplanted by non‐core species (Wilcoxon test, *p* < .01) (Figure S1A). In the diversity analyses, forager bees had significantly lower alpha diversity than nurse bees, suggesting that they harbored less rich and less even gut communities (Wilcoxon test, *p* < .05) (Figure 2a). In contrast, the betadisper analysis showed the longer distance of gut communities to the estimated centroid in forager bees according to Bray–Curtis dissimilarity (*p* < .001) (Figure 2b), indicating that they were more variable and dissimilar. This suggests that the gut community states in forager bees are not as tightly clustered as in nurse bees. Moreover, principal coordinate analysis (PCoA) measured with weighted UniFrac distances demonstrated that the gut community compositions of forager bees were widely dispersed compared to the tight clustering observed in nurse bees (Adonis, *R* ^2^ = .566; *p* < .001) (Figure 2c). These results suggest that forager bees have lower bacterial abundance and less stable gut communities than nurse bees.

### 3.2 Microbial characteristics of same‐aged worker bees with different tasks from a manipulated hive

**FIGURE 3:**
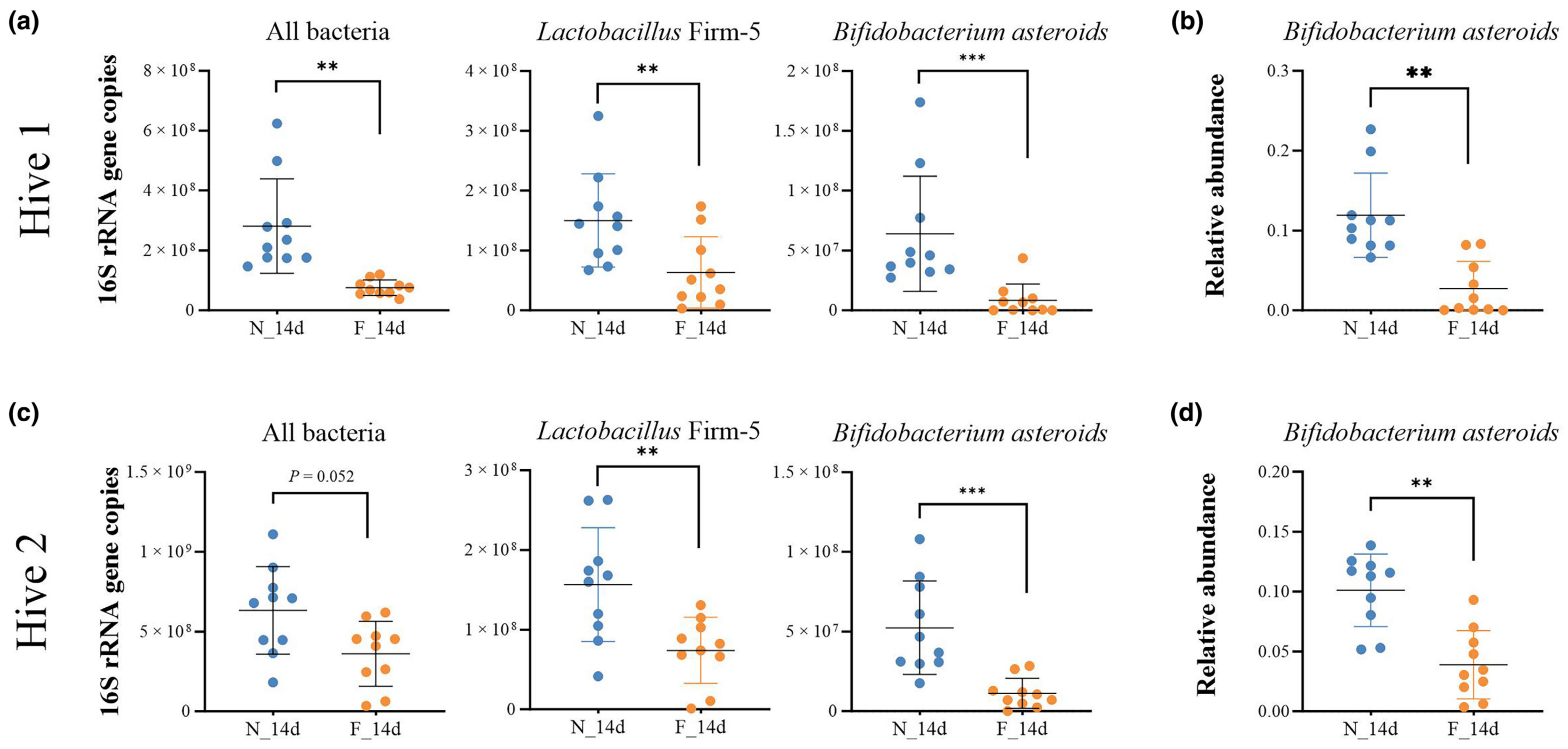
Honey bee gut microbiome composition in 14‐day‐old manipulated nurse (N_14d) and forager (F_14d) bees. Absolute (a, c) and relative (b, d) abundance of total and affected core bacterial members. Forager bees had lower total and core bacterial abundances. Significance was set at **p < .01, ***p < .001.

**FIGURE 4:**
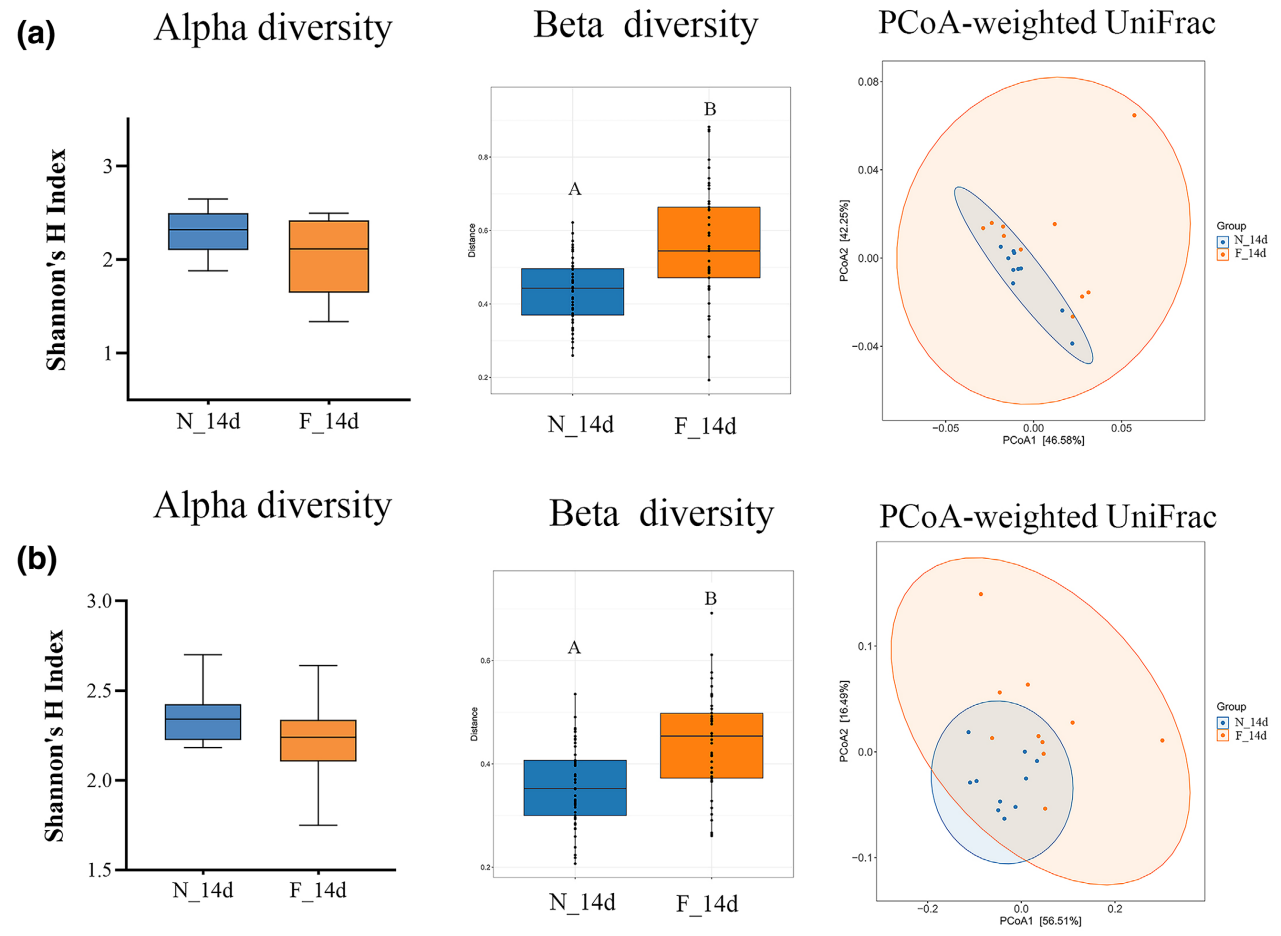
Honey bee gut microbiome composition in 14‐day‐old manipulated nurse (N_14d) and forager (F_14d) bees.

By manipulating the hive, we obtained 14‐day‐old worker bees with different behavioral traits. Similar to forager bees from the natural hive, those from the manipulated hive had a lower total abundance of bacteria and lower levels of the core species *L*. Firm 5 and *B. asteroides* compared to nurse bees from the manipulated hive (Wilcoxon test, *p* < .05) (Figure 3a). These results were consistent across both manipulated hives used in the experiment (Hive 1 and Hive 2) (Figure 3c). Additionally, compared to nurse bees of the same age, forager bees were more frequently colonized by non‐core species in Hive 1 (Wilcoxon test, *p* < .05) (Figure S1B). However, this change might depend on environmental factors, as it was less apparent in Hive 2 (Wilcoxon test, *p* > .05) (Figure S1B). Differences in the gut community structure were also evident in the relative proportions of different taxa. Notably, *B. asteroides* was the only core species that showed a significant difference in relative abundance between the nurses and foragers from the manipulated hives (Wilcoxon test, *p* < .01) (Figure 3b,d), emphasizing the importance of this bacterial species in the relationship between gut microbiota and social behavioral traits in honey bees. Although no significant difference was observed in alpha diversity (Wilcoxon test, *p* > .05) (Figure 4a,b) in two independent hives, beta diversity measured with Bray–Curtis dissimilarity was significantly dissimilarity in foragers in the manipulated hives (Wilcoxon test, *p* < .005) (Figure 4). Moreover, PCoA revealed a clear separation between the nurse and forager bees of the same age from manipulated hives (Adonis, *R* ^2^ = .194; *p* < .01) (Figure 4).

### 3.3 Microbial characteristics of honey bees fed different amounts of pollen

**FIGURE 5:**
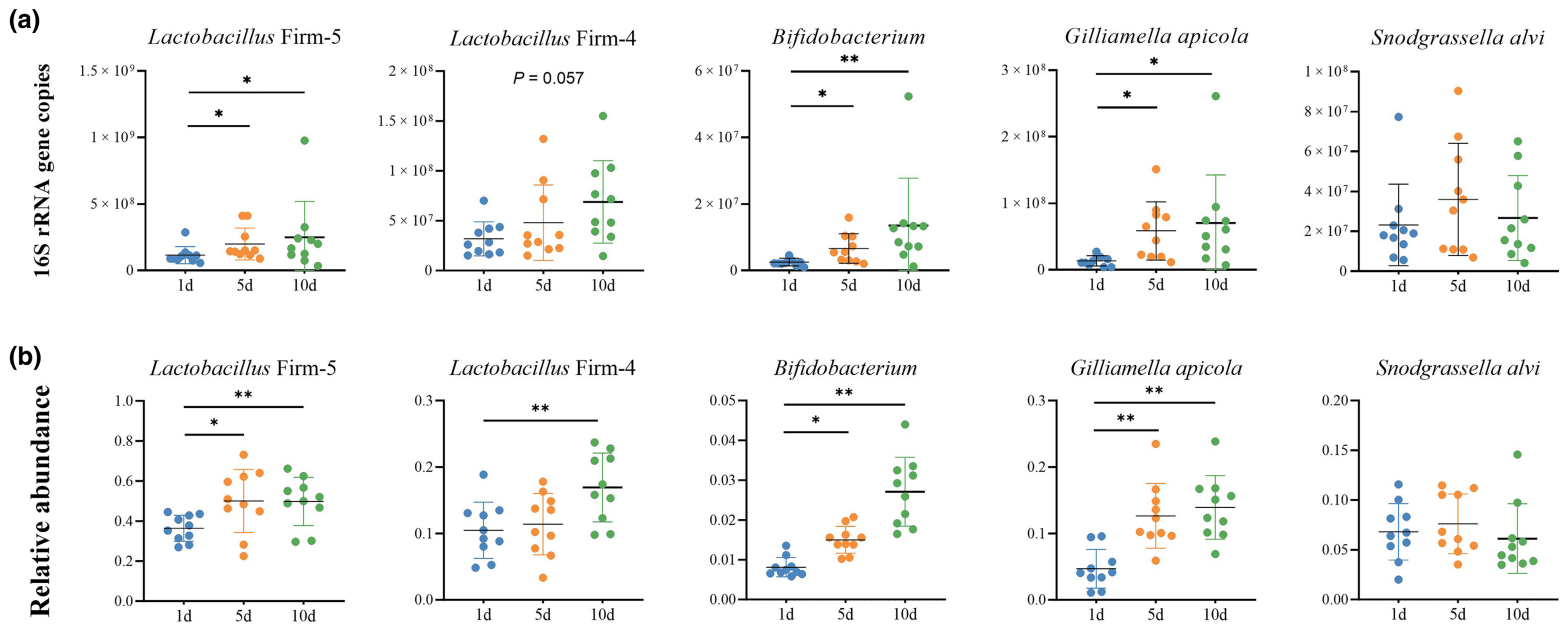
(a) Absolute and (b) relative abundance of the gut microbiome in honey bees fed varying amounts of pollen. Significance was set at *p < .05, **p < .01.

**FIGURE 6:**
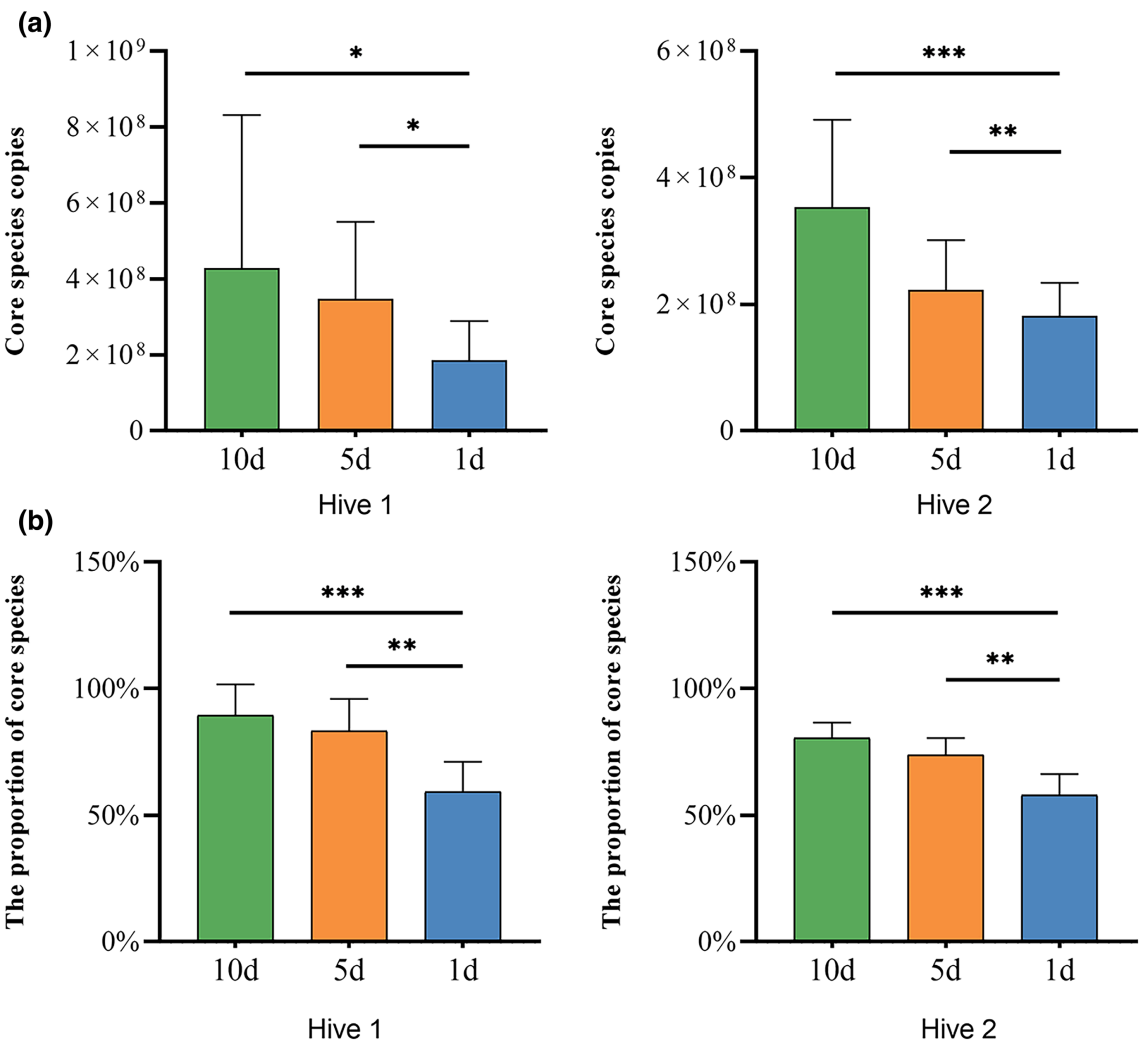
Absolute and relative abundance of all core bacterial species in the gut community of bees fed varying amounts of pollen. Significance was set at *p < .05, **p < .01, ***p < .001.

Our results indicated that diet is a key driving force shaping microbial diversity in honey bees. Notably, we determined that pollen consumption did not significantly affect the total bee gut microbial load (Wilcoxon test, *p* > .05) (Figure S2) but did promote the reproduction of the dominant core bacteria, except *S. alvi*, both in absolute and relative abundance (Wilcoxon test, *p* < .05) (Figure 5 and Figure S3). As pollen consumption increased, gut microbial alpha (measured with Shannon's index) and beta (measured with Bray–Curtis dissimilarity) diversity gradually decreased and showed clear separation according to the PCoA results (Wilcoxon test, *p* < .05) (Figure S4). Moreover, honey bees restricted from pollen feeding were more frequently colonized by non‐core species than unrestricted bees (Wilcoxon test, *p* < .05) (Figure 6).

## 4 DISCUSSION

Highly complex gut communities can change throughout their host's life, and their characteristics are typically regulated by external environmental factors and internal microbial interactions (Ellegaard & Engel, 2019). Furthermore, the gut microbiome can contribute to the adaptability of the host to the environment by mediating physiological and behavioral regulation (Archie & Tung, 2015), such as in stimulating cold tolerance (Koštál et al., 2016) or increasing competitive mating (Jia et al., 2021). In this study, we evaluated the gut bacterial characteristics of honey bees with the highly representative division of labor and highlighted the potential link between the gut microbiome and behavioral tasks in honey bees. Our study supports the idea that eusocial communities typically form a common gut microbiome because of shared social environments (Kapheim et al., 2015; Kwong et al., 2017). All nine bacterial species clusters were identified, comprising five core and four non‐core species, with five core species clusters dominating the bee guts in all samples. Considering the frequent trophallactic interactions between nurse and forager bees, such as during nectar transport and processing, their similar microbiome taxa may be due to recurrent re‐inoculation (Crailsheim, 1998; Kapheim et al., 2015; Powell et al., 2014). However, there were remarkable differences in bacterial abundance and community structure between worker bees performing different behavioral tasks, despite having a similar microbiome taxon. Kešnerová et al. (2020) demonstrated that nurse and winter bees harbor more abundant gut bacteria than forager bees. Additionally, the average population replication of core phylotypes was found to be lower in 24‐day‐old honey bees than in 10‐day‐old bees (Ellegaard & Engel, 2019). We reached similar conclusions by calculating the absolute abundance of gut bacteria in natural worker bees. Our data confirmed that forager bees had fewer gut microbes than nurse bees, especially the core species *L*. Firm 5, *L*. Firm 4, and *B. asteroides*. Furthermore, a lower absolute load in forager bees was associated with lower relative composition in our study, which is somewhat consistent with the findings of a previous study (Kapheim et al., 2015; Vernier et al., 2024).

Nurse bees have been shown to transition to foragers as they age and lose their nutrient stores (Smith et al., 2022). To exclude the interference of age‐related physiological influences, we examined the gut microbiome of nurse and forager bees of the same age using a manipulated hive. Similar to the results of our previous experiments, a lower gut microbial load, comprising *L*. Firm 5 and *B. asteroides*, was observed in foragers from manipulated colonies compared to that in nurse bees. However, a lower level of *L*. Firm 4 colonization was not widespread in foragers and did not occur in foragers from manipulated hives. Of the five core species, only *B. asteroides* showed a lower relative abundance in worker bees from manipulated hives. Jones et al. (2018) conducted an experiment using a single‐cohort colony where worker bees of the same age performed different tasks; however, the differences in microbial characteristics between the nurse and forager bees in their manipulated hives were more similar to those in our natural bee colonies than those in our manipulated hives. Specifically, they found that foragers had a lower relative abundance of core species (*L*. Firm 5, *L*. Firm 4, and *B. asteroides*), similar to that of our natural bees. However, only *B. asteroides* showed a lower relative abundance in our foragers from manipulated hives. These discrepancies may be due to differences in experimental design between the single‐cohort colony and our manipulated hives. The single‐cohort colony was first established by Robinson (1992), and its composition was characterized by only a queen and 0‐ to 2‐day‐old workers. Under such conditions, workers can complete the social division of labor within a few days, with some individuals exhibiting foraging behavior as early as 5 days old, which is 2 weeks earlier than normal. Instead, we adopted a moderate approach by referencing Amdam's manipulated hive method to obtain nurse and forager bees (Amdam et al., 2005), in which nurse bees rapidly establish a division of labor due to the partial removal of older foragers. Theoretically, the division of labor is more abrupt, thorough, and efficient in single‐cohort colonies; therefore, the difference in physiological conditions between manipulated nurse and forager bees in single‐cohort colonies should be more pronounced than that in our manipulated hive. In our study, hive manipulation simulated the early stages of the division of labor, while a single‐cohort colony can be seen as representing a completely developed change. This may explain why the differences in microbial characteristics between the manipulated nurse and forager bees in the single‐cohort colony were more similar to those of our natural bee colonies than to those of our manipulated hives. However, the full impact of complete destruction and restructuring in a single‐cohort colony is unknown. Furthermore, in the single‐cohort method, young bees tend to establish atypical microbial communities of non‐core species or highly skewed microbial compositions because they have no access to oral trophallactic interactions with natural nurse bees (Powell et al., 2014). This biological context should be considered, particularly in research focusing on microbial characteristics.

Overall, there were marked differences in the gut microbiota of nurse and forager bees, both in terms of total bacterial abundance and the relative abundance of individual species. Moreover, we speculated that the microbial changes increase gradually with the transition from nurse to forager. In the manipulated hives, workers in the early transition stage exhibited marked changes in both the absolute and relative abundances of *B. asteroides*. In comparison, significant variation in *L*. Firm 5 was only reflected in absolute abundance but not in relative abundance. However, the three primary core species showed significantly lower absolute and relative abundances in natural foragers than in nurse bees. In this study, gut community surveys of forager and nurse bees from manipulated and natural hives suggested that *B. asteroides* abundance changes first, followed by that of *L*. Firm 5 and *L*. Firm 4. Yet, uncertainty remains regarding the driving factors of distinct gut bacterial characteristics between nurses and foragers. Consistent with previous speculations (Kešnerová et al., 2020), we concluded that diet may be a major contributing factor to differences in gut microbiota. Nurse bees typically consume considerable amounts of pollen, whereas foragers prefer nectar and honey. Previous studies have shown that pollen consumption can increase the total bacterial abundance and individual phylotype abundance in bee guts under laboratory conditions (Kešnerová et al., 2020; Ricigliano et al., 2017). Notably, Ricigliano et al. (2017) found that only two core species, *L*. Firm 5 and *B. asteroides*, changed, whereas Kešnerová et al. (2020) found that all five core phylotypes increased with pollen consumption. This discrepancy may be due to the difference in pollen feeding time between the two studies. The food limitation experiment in the current study demonstrated that pollen can promote colonization by core microbial communities.

Uncertainty remains about the mechanisms that ensure the stability of the three core species and why *B. asteroides* shows the highest sensitivity to behavioral tasks among all core species. Honey bees have a relatively simple gut microbial community dominated by five core species clusters and four rare non‐core species clusters. Non‐core species are comparatively less numerous and less prevalent in the guts of honey bees and the hive environment (Kwong & Moran, 2016). Among the five core species, *G. apicola* and *S. alvi* are considered more stable, especially *S. alvi*, as they predominantly colonize the physically restricted epithelial lining of the ileum (Kešnerová et al., 2020; Li et al., 2022; Martinson et al., 2012). In this study, these species exhibited greater consistency between the two worker bee types. Recent evidence has revealed that *S. alvi* is adapted to a specific metabolic niche in the gut that depends on host‐derived nutritional resources rather than food in the gut (Quinn et al., 2024). The relatively close evolutionary relationships among *L*. Firm 5, *L*. Firm 4, and *B. asteroides* have resulted in similar biochemical characteristics. These three species are facultative anaerobes and gram‐positive bacteria that share common specialized localizations in the rectum (Bottacini et al., 2012; Kawasaki et al., 2006; Killer et al., 2014; Olofsson et al., 2014), where they may function in the reabsorption of fecal waste (Kwong & Moran, 2016). Additionally, *L*. and *B. asteroides* in the bee gut facilitate the digestion of plant polysaccharides (Engel et al., 2012; Zheng et al., 2019) and are therefore considered fermentative bacteria (Kwong & Moran, 2016). *Bifidobacteria*, in particular, exhibit remarkable polysaccharide degradation abilities due to their possession of numerous genes related to this function, such as glycoside hydrolase families, compared to other species (Zheng et al., 2019). This evidence may explain why *B. asteroides* are the most sensitive to behavioral tasks among all core species. The previous research found that the relative abundance of *Lactobacillus* and *Bifidobacterium* are most prone to fluctuations. For example, Hotchkiss et al. (2022) summarized recent studies investigating the effects of pesticides on honey bee gut microbiota and determined that shifts in core microbial species were most common among *Lactobacillus* and *Bifidobacterium* spp. In light of our results, a reasonable inference is that the changes in the abundance of these microbial communities may be attributed to variations in dietary intake interfered with by a bunch of toxicants or parasites or something else.

Recently studies have found that gut microbes can regulate the social division of labor in honey bees, such as foraging onset (Vernier et al., 2024) and intensity (Liberti et al., 2023). Here, we reveal a shift from feeding on pollen at the nurse stage to feeding on nectar at the forager stage is a driving factor in gut microbial diversity. Based on the findings of the current study and previous reports, we propose a hypothesis for the possible interaction between the gut microbiome and behavioral tasks in honey bees.

## AUTHOR CONTRIBUTIONS

**Kang Wang:** Conceptualization (lead); methodology (lead); writing – original draft (lead). **Ming Zheng:** Conceptualization (supporting); methodology (equal); software (lead). **Minqi Cai:** Data curation (lead); formal analysis (supporting). **Yi Zhang:** Project administration (equal); writing – original draft (supporting). **Yuanchan Fan:** Data curation (supporting); software (supporting). **Zheguang Lin:** Formal analysis (supporting); investigation (supporting); project administration (equal). **Zhi Wang:** Resources (lead); supervision (supporting). **Qingsheng Niu:** Resources (equal); supervision (equal); writing – original draft (equal). **Ting Ji:** Funding acquisition (lead); project administration (lead); writing – original draft (equal).

## FUNDING INFORMATION

The financial support was granted by the Young Elite Scientists Sponsorship Program by Yangzhou University (KW), the National Natural Science Foundation of China (32272935, ZL), and the Earmarked Fund for Modern Agro‐industry Technology Research System (CARS‐44, TJ).

## CONFLICT OF INTEREST STATEMENT

The authors declare no competing financial interest.

## Supporting information


Figures S1–S4.

## Data Availability

The raw sequence data reported in this paper have been deposited in the Genome Sequence Archive in the National Genomics Data Center, China National Center for Bioinformation / Beijing Institute of Genomics, Chinese Academy of Sciences (GSA: CRA014520, CRA014555, and CRA014557) that are publicly accessible at https://ngdc.cncb.ac.cn/gsa.
