# Supplementary material for: Possible interactions between gut microbiome and division of labor in honey bees
Source: Ecol Evol. 2024 Aug 27;14(8):e11707. doi: 10.1002/ece3.11707 (PMC11348130; doi:10.1002/ece3.11707)
Supplement: Supplementary file 1 — Figures S1–S4. [file ECE3-14-e11707-s001.docx]

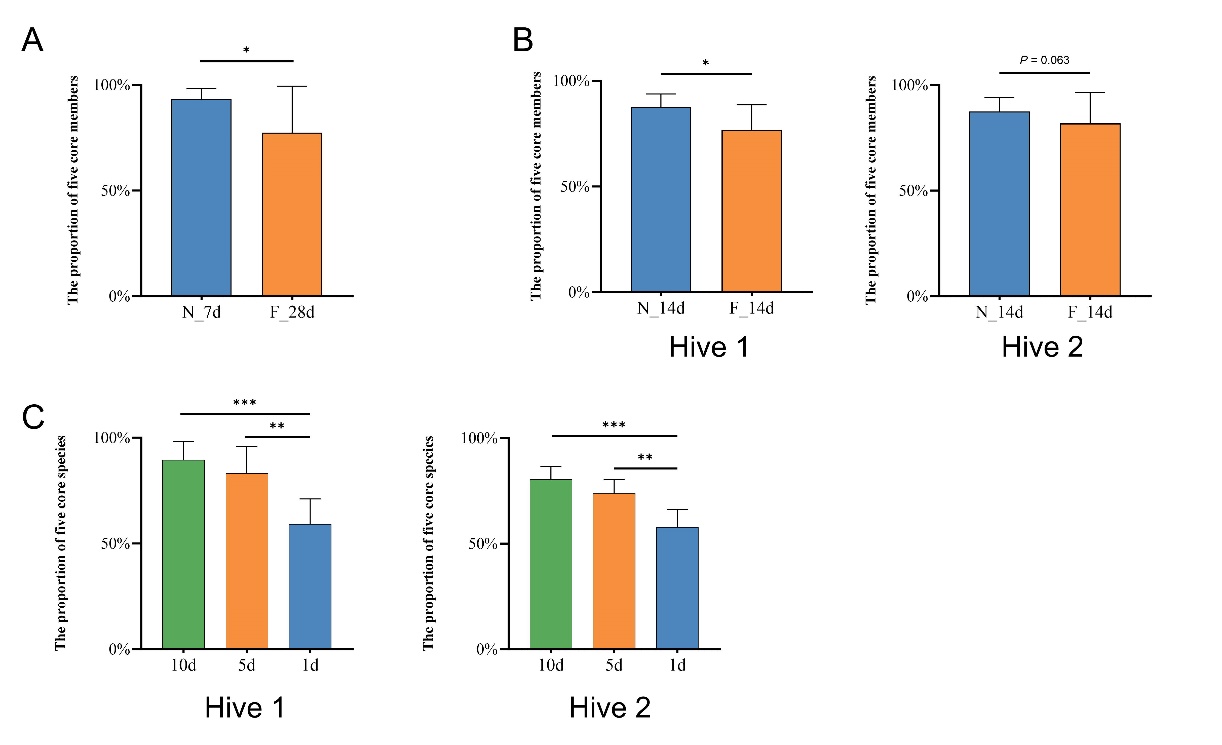


Fig. S1. Proportions of all core bacterial species in the bee gut community of (A) natural nurse (N) and forager (F) bees, (B) nurse and forager bees of the same age from manipulated hives, and (C) honey bees with access to varying amounts of pollen (*P* < 0.05).


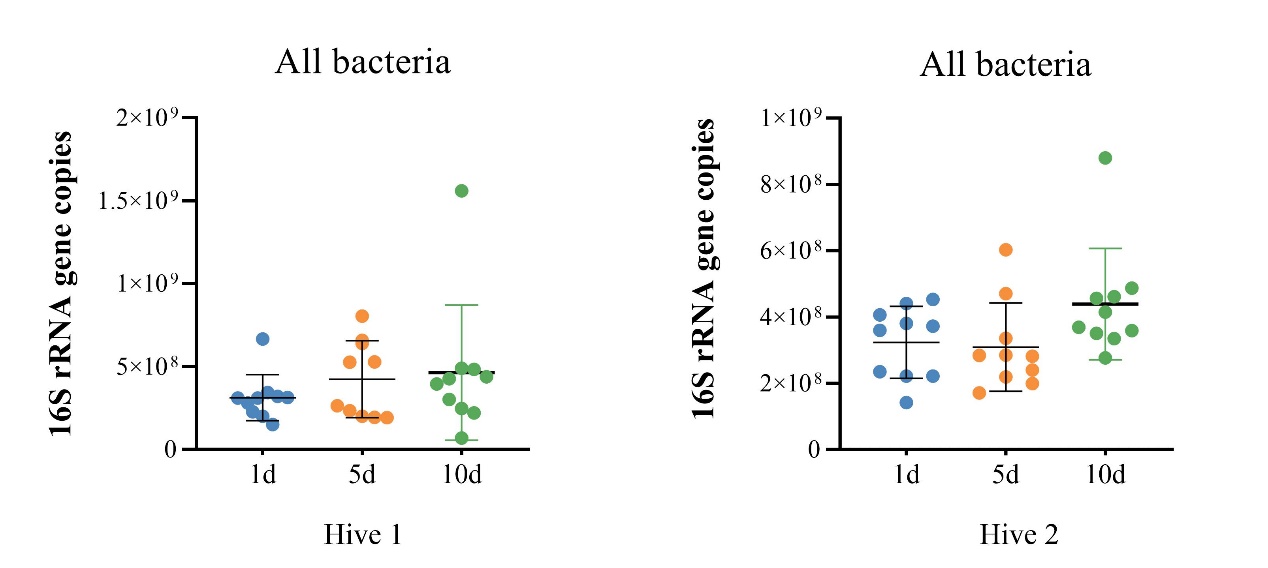


Fig. S2. Gut microbiome abundance in honey bees fed varying amounts of pollen.


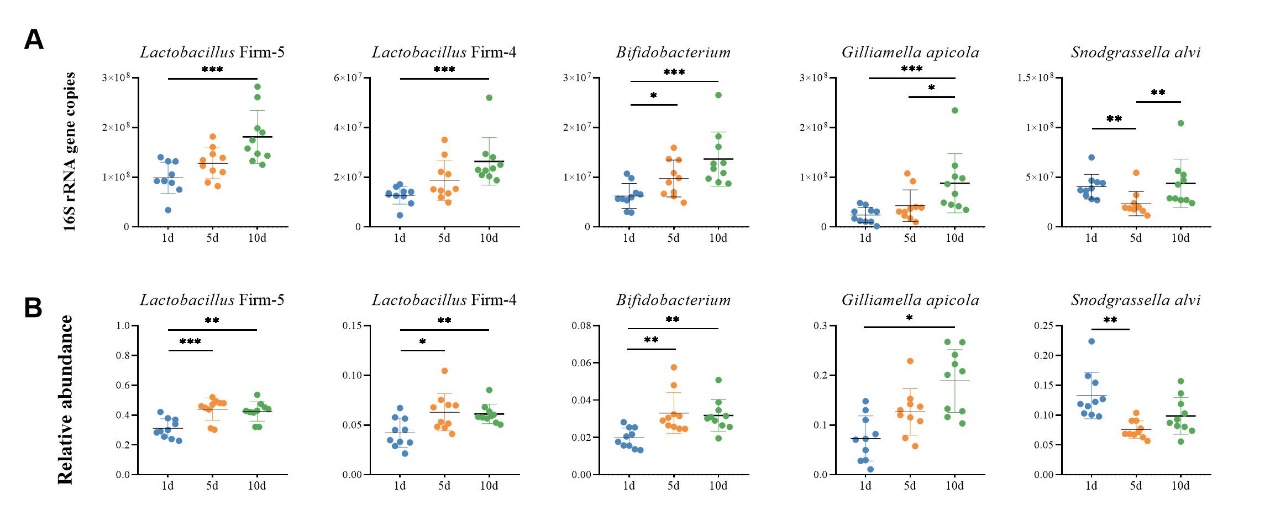


Fig. S3. (A) Absolute and (B) relative abundances of the gut microbiome in honey bees in hive 2 that were fed varying amounts of pollen.


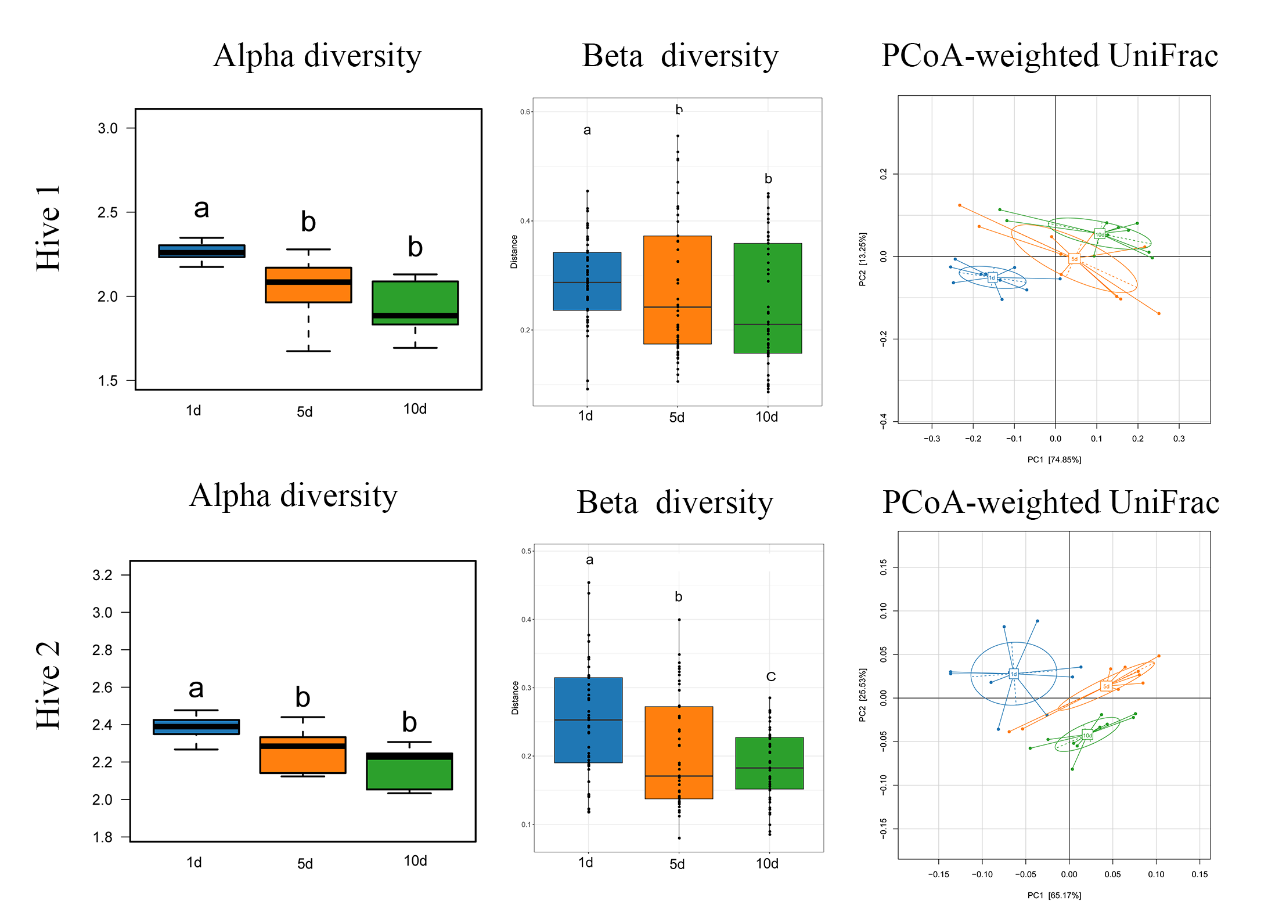


Fig. S4. Gut microbiome composition of honey bees fed varying amounts of pollen.
